# Experimental Research Progress on Gas–Liquid Flow and Heat Transfer Characteristics in Micro Pulsating Heat Pipes

**DOI:** 10.3390/mi17010037

**Published:** 2025-12-29

**Authors:** Jun Chen, Hao Tian, Wanli Xu, Huangdong Guo, Chao Wang, Jincheng Gu, Yichao Cao

**Affiliations:** 1 School of Petroleum and Natural Gas Engineering, School of Energy, Changzhou University, Changzhou 213164, China; 2 School of Intelligent Manufacturing and Smart Transportation, Suzhou City University, Suzhou 215104, China; 3 Valiant Co., Ltd., Yantai 264006, China; 4 Jiangsu Key Laboratory of High Performance Fiber Composites, Changzhou 213135, China; 5 JITRI-PGTEX Joint Innovation Center, Changzhou 213135, China

**Keywords:** MPHP, visualization experiment, fluid dynamics behavior, heat transfer characteristics

## Abstract

As the power density of microelectronic devices and components continues to increase, thermal management has become a critical bottleneck limiting their performance and reliability. With its advantages of effective heat dissipation, no need for external power, and good safety, the micro pulsating heat pipe (MPHP) exhibits unique application advantages and enormous development potential when compared to other cutting-edge thermal management solutions, such as embedded microchannel cooling technology, which has complicated manufacturing processes and is prone to leakage, or thermoelectric material cooling technology, which is limited by material efficiency and self-heating. However, a pulsating heat pipe (PHP) is vulnerable to the combined impacts of several elements (scale effects, wall effects, and interfacial effects) at the micro-scale, which can lead to highly variable heat transfer characteristics and complex two-phase flow behavior. There are still few thorough experimental reviews on this subject, despite the fact that many researchers have concentrated on the MPHP and carried out in-depth experimental investigations on their flow and heat transmission mechanisms. In order to provide strong theoretical support for optimizing the design of the MPHP cooling devices, this paper reviews previous experimental research on the MPHP with the goal of thoroughly clarifying the mechanisms of gas–liquid two-phase flow and heat/mass transfer within them. The definition of MPHP is first explained, along with its internal energy transmission principles and structural features. The motion states of gas–liquid two-phase working fluids in the MPHP from previous experimental investigations are then thoroughly examined, highlighting their distinctive flow patterns and evolution mechanisms. Lastly, the variations in thermal performance between different kinds of MPHPs are examined, along with the factors that affect them.

## 1. Introduction

Integrated circuits have developed according to a steady trend for decades: the size of electronic components keeps becoming smaller while the number of transistors nearly doubles every two years. As a direct result of this trajectory, chip thermal power density has skyrocketed, leading to ever-increasing integration and downsizing [1,2]. For instance, the most recent generation of chips’ heat flux density has already surpassed 800 W/cm^2^ [3], which poses a major risk to the stability and safety of both the chips and the system as a whole. However, due to their low efficiency and need for external power sources, conventional cooling technologies—such as air cooling [4], liquid cooling [5], and conventional heat pipes [6,7]—are unsuitable for the thermal management requirements of small electronic components. Thus, the development of a new, highly effective cooling technology for small electronic components is urgently needed.

In the field of thermal management for high heat flux density electronic components, current research focuses on embedded microchannel cooling technology, thermoelectric material cooling technology [8], and micro pulsating heat pipe (MPHP for short) cooling technology. While embedded microchannel cooling technology offers high thermal efficiency, it suffers from complex manufacturing processes, susceptibility to leakage, and poor safety performance [9]. Thermoelectric material cooling technology, based on the Peltier effect, achieves solid-state “heat pump” transport through electrical current. This technology requires no mechanical moving parts, offering advantages such as compact structure, precise temperature control, and rapid response. However, it incurs high material costs (relying on rare elements like bismuth and tellurium), exhibits a low cooling coefficient of performance (COP), and notably generates significant self-heating, necessitating efficient cooling systems [10]. The MPHP’s cooling technology offers unique advantages, including superior heat dissipation, compact structure, no external power requirement, and high safety. It demonstrates broad application potential across numerous fields [11,12,13]. Notably, recent advances in device-level thermal management have revealed critical coupling relationships between heat transfer, interface effects, and device performance [14]. Consequently, thermoelectric material cooling and MPHP cooling can form a clear upstream-downstream synergistic relationship: high-efficiency thermoelectric materials can reduce waste heat generation at the source, while the MPHP rapidly dissipates existing waste heat. Their coupled application enables end-to-end optimization from heat generation to waste heat dissipation.

In summary, MPHPs occupy an irreplaceable technological niche in high heat flux density thermal management due to their unique passive self-oscillating two-phase heat transfer mechanism. However, comprehensive reviews on MPHPs remain scarce. This paper systematically summarizes existing experimental work on flow and heat transfer in MPHPs, further enriching research in micro-scale flow and heat transfer. It provides valuable references and guidance for subsequent experimental studies.

**Figure 1:**
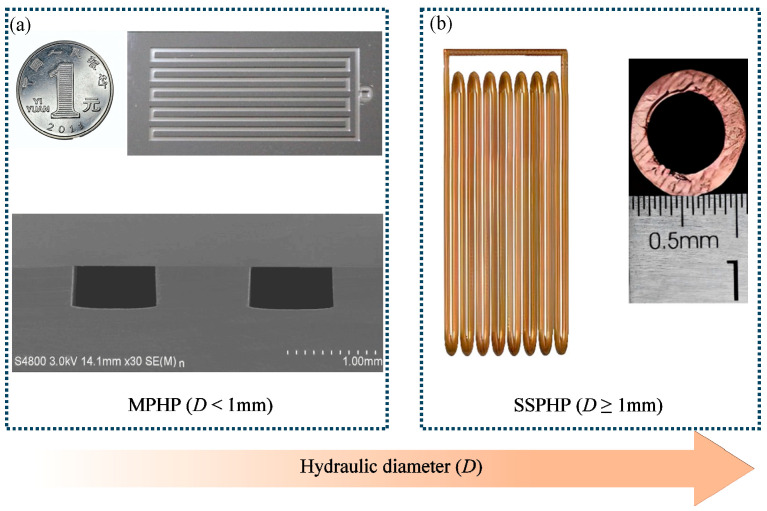
Size classification basis of PHP: (**a**) MPHP; (**b**) SSPHP.

The channel dimensions are the primary distinction between the MPHP and the small-scale pulsating heat pipe (SSPHP for short), as illustrated in Figure 1. The SSPHP usually has a hydraulic diameter of more than 1 mm, whereas the MPHP has a much smaller hydraulic diameter that frequently approaches the micrometer level. In other words, a pulsating heat pipe (PHP for short) with a hydraulic diameter of less than 1 mm is usually referred to as MPHP [12].

**Figure 2:**
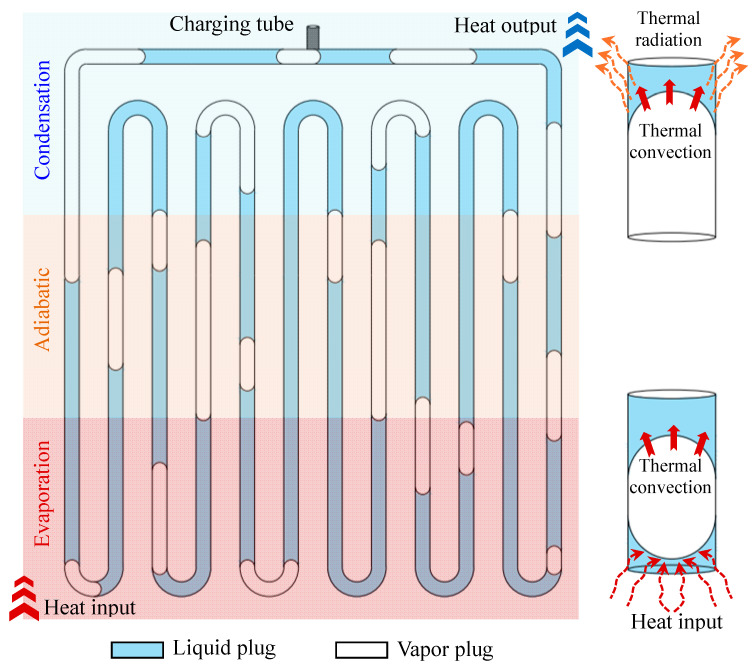
The operation principle diagram of the MPHP.

A number of dynamically connected processes, such as bubble nucleation, expansion, coalescence, contraction, rupture, and the evaporation and condensation of liquid layers, are involved in the working concept of the MPHP. Based on their operational characteristics, the MPHP’s tube segments are separated into evaporation, adiabatic, and condensation sections, as illustrated in Figure 2. Initially, surface tension greatly affects the working fluid inside the pipe [15], which is limited by its small dimensions. This results in the formation of randomly distributed gas and liquid plugs. The working fluid absorbs heat in the evaporation section during operation, changing its phase and creating bubbles. The working fluid is propelled into the condensation section by the abrupt local pressure rise caused by these rapidly expanding bubbles. When the condensing portion cools, the bubble/gas plug compresses and bursts, lowering pressure and creating working fluid reflux. Heat transfer from the evaporation section to the condensation section is made possible by the refrigerant’s periodic oscillatory motion within the MPHP, which is driven by pressure differentials between tube sections and pressure imbalances between neighboring tubes [16].

## 2. Experimental Research Methods

The oscillatory behavior of working fluids within PHP is affected by additional elements under micro-scale effects [17,18], leading to flow characteristics that are very different from those seen in SSPHPs. Clarifying the heat transmission mechanisms of PHP requires a deep comprehension of the flow properties of both gas and liquid phases. As a result, researchers have used a variety of techniques to carry out a number of experimental investigations on the flow properties of gas–liquid two-phase working fluids.

**Figure 3:**
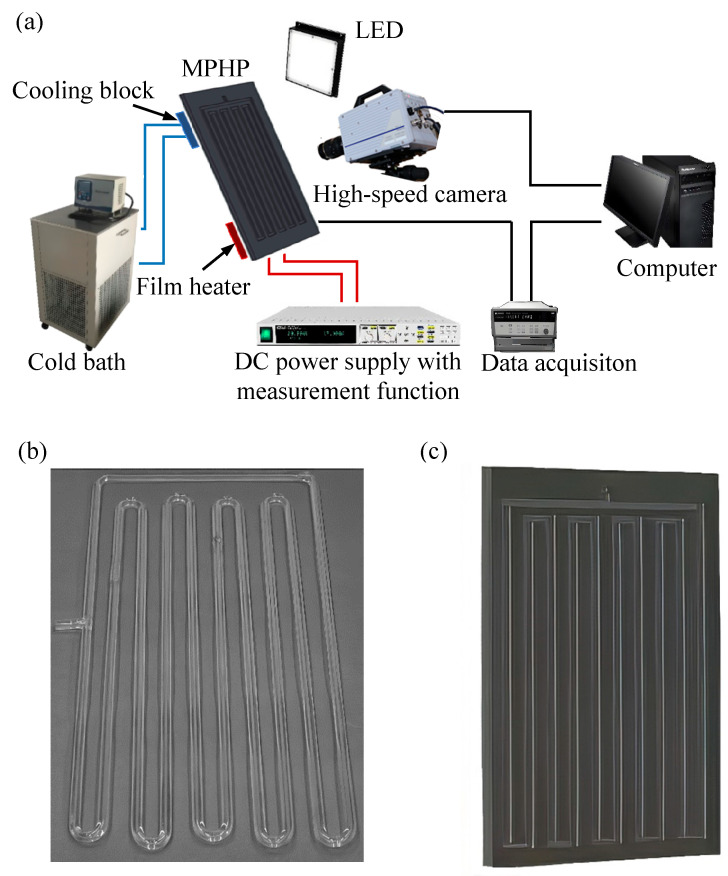
High-speed camera technology and its MPHP: (**a**) high-speed camera technology experimental system, (**b**) glass tube PHP, and (**c**) silicon substrate PHP.

Cattani et al. [19] and Iwata et al. [20] have used wavelet analysis to look into the working fluid’s pulsation frequency characteristics and infrared technology to see how tubular the MPHP operates. Infrared imaging is mostly used as a supplemental visualization technique [21] because it is unable to directly visualize the fluid flow events within PHP. Furthermore, Kim et al. [22] used laser-induced fluorescence techniques and added a fluorescent dye (rhodamine B) to the working fluid, ethanol, to study the distribution of heat flow in asymmetric MPHPs. Nevertheless, the regular flow of the working fluid is unavoidably disrupted when fluorescent dye is added to the pipe, resulting in inevitable experimental mistakes. The high-speed camera technique depicted in Figure 3a is currently the most used visualization method. This technique mainly examines a flat plate pulsating heat pipe (FPPHP) [23], which is made by sealing high-thermal-conductivity substrate materials (silicon or metal) with clear glass coverings, or a PHP made of transparent materials (such as quartz glass) in tubular shapes [24]. During startup and quasi-steady-state operation, high-speed cameras are used to monitor and document the flow behavior, flow patterns, and their evolutionary patterns of the working fluid within the PHP.

## 3. Experimental Study on Flow Characteristics of the MPHP

### 3.1. Flow Motion Patterns of Working Fluid in the MPHP

**Figure 4:**
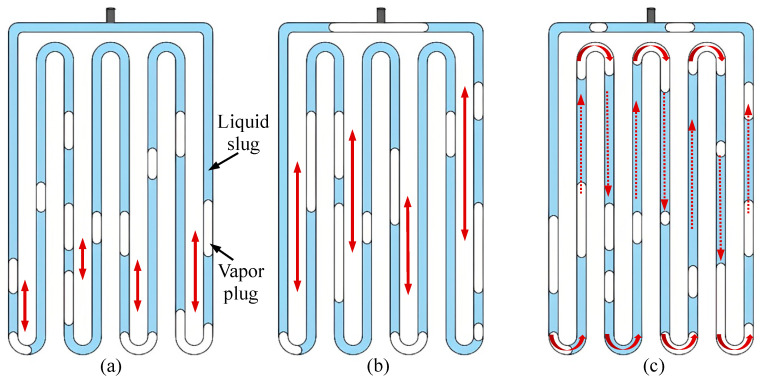
MPHP motion mode: (**a**) small oscillation (SO); (**b**) large oscillation (LO); and (**c**) circulation flow (C). Noted, the one-way arrow indicates the flow direction, and the two-way arrow indicates the oscillation amplitude.

As shown in Figure 4, the working fluid motion patterns within the MPHP are mainly divided into three categories: small oscillations (SO), large oscillations (LO), and circulation flow (C). Liquid and vapor plugs that randomly oscillate within a constrained range along the tube are the manifestation of SO. Long-distance, directionally specified reciprocating motion is initiated during LO by a large rise in the amplitude of liquid and vapor plug oscillations [25]. C can also happen in well-designed PHP [26]. Similarly to conventional heat pipes or pump-driven water circulation systems, the working fluid in this case stops reciprocating action and instead runs continuously along the heat pipe circuit.

**Figure 5:**
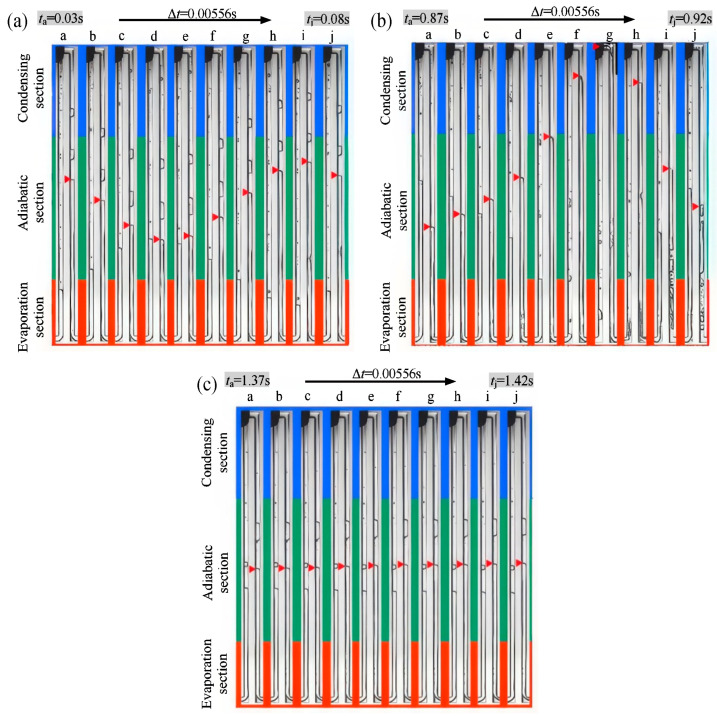
MPHP motion mode: (**a**) SO, (**b**) LO, and (**c**) stagnation. The image is sourced from the PhD dissertation of the corresponding author, Chao Wang [28]. Noted, the red arrow points to the real-time position of the gas-liquid interface.

Additionally, the pushing power produced by phase transition is occasionally insufficient to maintain orderly flow across the whole closed loop due to increased surface tension and flow resistance within PHP at the micro-scale. As a result, internal circulation in some MPHPs disappears, and during the alternating cycles of LO and SO, there is a temporary standstill phenomenon. No discernible circulation was found in the research by Wang et al. [25] and Liu et al. [27] on the use of acetone in rectangular MPHPs. Rather, they found short stagnation at the transition between the two states (Figure 5c), LO (Figure 5b), and SO (Figure 5a). The results of the study further show that stagnation is essentially a dynamic interaction between internal resistive forces (surface tension and viscosity) and the phase-change driving force within PHP. The driving force may hardly overcome resistance (SO) with low heat loads, which frequently results in stagnation because of energy dissipation. Localized drying inside the tube section and “overshoot” and rebound in bubble dynamics can also cause stagnation at medium to high heat loads during times of substantial oscillation within the tube (LO). Nevertheless, the working fluid is forced to reenter either SO or LO as energy builds up inside the tube and reaches a critical threshold.

According to current studies [29], one important aspect affecting the pipe’s flow patterns is the amount of input power. High-speed cameras were used by Qu et al. [30] to visualize a silicon-based MPHP with a hydraulic diameter of 394 μm and a trapezoidal cross-section. They observed the following phenomenon: The working fluid flow inside the tube started displaying thermosiphon characteristics at an input power of 2.4 W. At this point, condensate flowed downward while vapor in the tube flowed upward along the channel wall. The working fluid showed clear oscillatory activity as the input power rose to 4.6 W. The fluid inside the tube then progressively adopts self-sustained oscillation as its main motion pattern. The oscillation frequency dramatically increases when the input power reaches 6.9 W. The initial oscillatory motion is gradually replaced by the C, and the working fluid can even cross bends to enter neighboring tubes.

**Figure 6:**
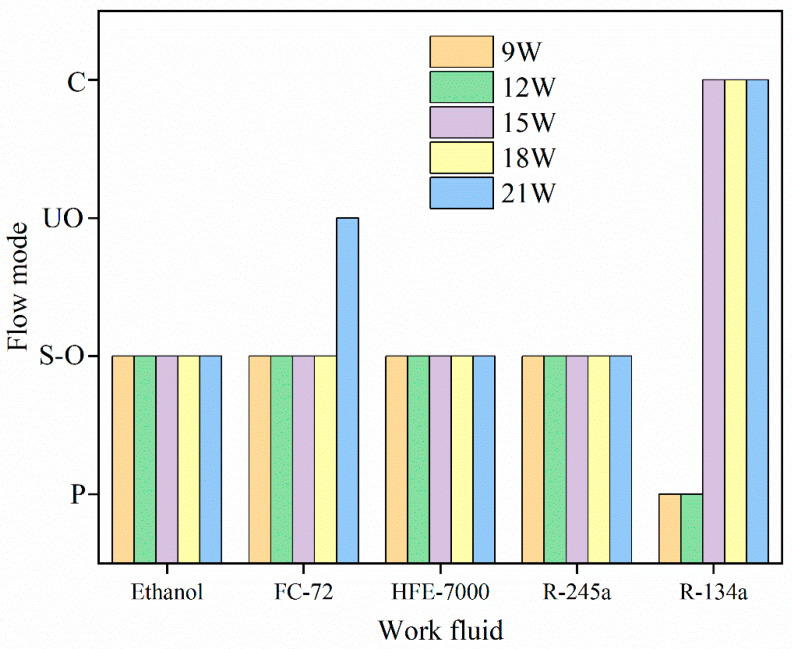
Flow motion patterns of various working fluids at different input powers; the data analysis is from the literature [31].

In addition to input power, working fluid parameters and fill ratio also influence the flow patterns within the tube. Using five distinct working fluids, Kim et al. [31] performed visualization investigations on a rectangular cross-section MPHP with a hydraulic diameter of 667 μm. Flow motion patterns of various working fluids at different input powers under 50% fill ratio settings are shown in Figure 6. It was discovered that stable oscillating flow (S-O), pulsing flow (P), and unstable oscillating flow (UO) with stagnation phenomena were seen as the input power rose steadily. However, only the MPHP with R-134a as the working fluid showed circulating flow (C). This is due to R-134a’s superior working fluid characteristics, which include low latent heat (allowing fluid motion with minimal evaporation heat), low dynamic viscosity (producing minimal flow resistance), low surface tension (producing minimal flow resistance), and high (d*P*/d*T*)_sat_ (allowing a large pressure differential between the evaporator and condenser). These characteristics successfully overcome obstacles and advance circulation. On the other hand, working fluids with poorer parameters—like acetone—find it difficult to reach a steady circulation.

Using HFE-7100 as the working fluid at a fill ratio of 31–72%, Sun et al. [32] carried out visualization experiments using high-speed camera technology on a trapezoidal cross-section MPHP with a hydraulic diameter of 357 μm. The MPHP’s oscillatory motion can be roughly divided into two phases: the large-amplitude oscillation phase (LAOP for short) and the small-amplitude oscillation phase (SAOP for short). Furthermore, transient circulation flow was noted. In particular, the duration of the “LAOP” decreases while the equivalent “SAOP” period increases as the fill ratio rises to 65% fill. On the other hand, the “LAOP” duration lengthens when the fill ratio drops (53% and 40% fill), increasing the likelihood of circulation.

Naturally, using the proper fill ratio, working fluid parameters, and input power does not ensure that circulation will occur. Qu et al. [11,30,33,34] and Sun et al. [32,35] carried out a visualization investigation on uniform MPHPs with different hydraulic diameters under the same operating conditions using high-speed camera technology. Within the symmetric channel MPHP, they found a critical hydraulic diameter of 352 μm; PHPs less than this critical diameter cannot attain circulation. This happens because PHP’s flow resistance is too high below the critical diameter, necessitating more heating power to start circulation. The pipe portion frequently dries up before reaching circulation.

**Figure 7:**
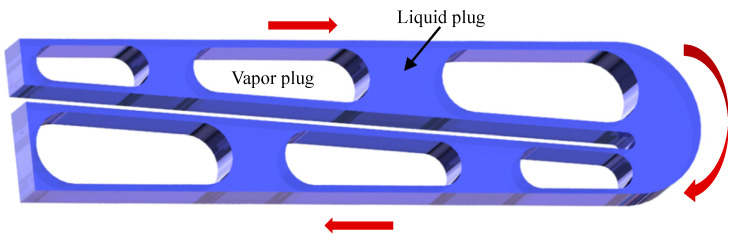
Variable diameter design of microchannels. Noted, the one-way arrow indicates the flow direction.

Interestingly, an asymmetric channel may also encourage the production of circulation inside the tube. Variable diameter design is one of the commonly used methods, as illustrated in Figure 7. For example, Sun et al. [36] looked into how channel shapes affected working fluid flow characteristics in MPHPs. In MPHPs with gradient cross-sections, they found transient directional circulation. The duration of this event increased as the input power increased. MPHPs with constant cross-sections did not exhibit this effect. The article explains this phenomenon by stating that the gradient cross-sectional structure produces additional capillary driving forces.

**Figure 8:**
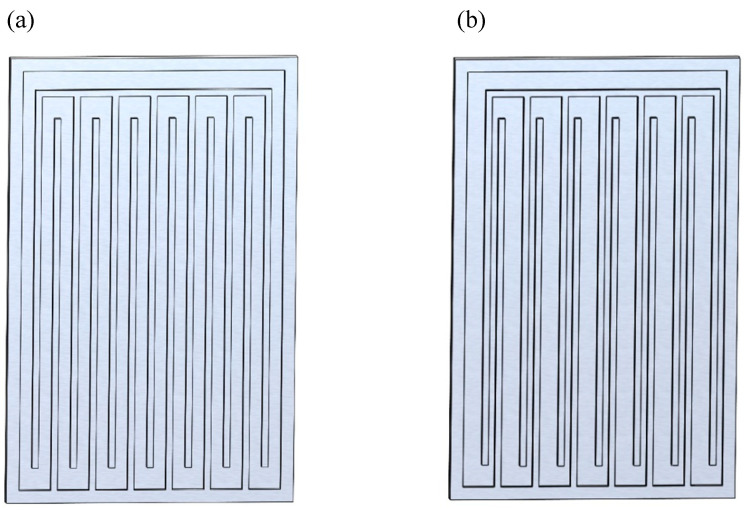
Uneven channel spacing design of microchannels: (**a**) symmetric MPHP; (**b**) asymmetric MPHP.

Uneven channel spacing design (shown in Figure 8) is also a commonly used method. For example, Kim et al. [22,37] performed visualization tests on symmetric and asymmetric (dual-diameter channel) MPHPs. They found that asymmetric channels encourage the production of circulation within the pipes in contrast to symmetric arrangements. Additionally, it was shown that two important variables in producing circulation inside asymmetric MPHPs are diffusion and the creation of interface deformations.

### 3.2. Flow Patterns and Their Evolution in MPHPs

In MPHPs, the motion condition within the pipe frequently affects the working fluid’s evolution and flow pattern. The efficiency of energy and mass transport within the heat pipe is determined by the dynamic relationship between these two parameters, which both constrain and couple with one another. Furthermore, the working fluid’s phase transition mechanism inside microchannels is extremely intricate. The channel walls significantly restrict phenomena, including bubble nucleation, expansion, and coalescence. Individual bubbles may even obstruct the channel because their diameters are similar to the channel’s dimensions [38], adding more unpredictability and instability to the flow and heat transfer processes [11,30]. Researchers from all across the world have used visualization approaches for observation in order to address issues.

**Figure 9:**
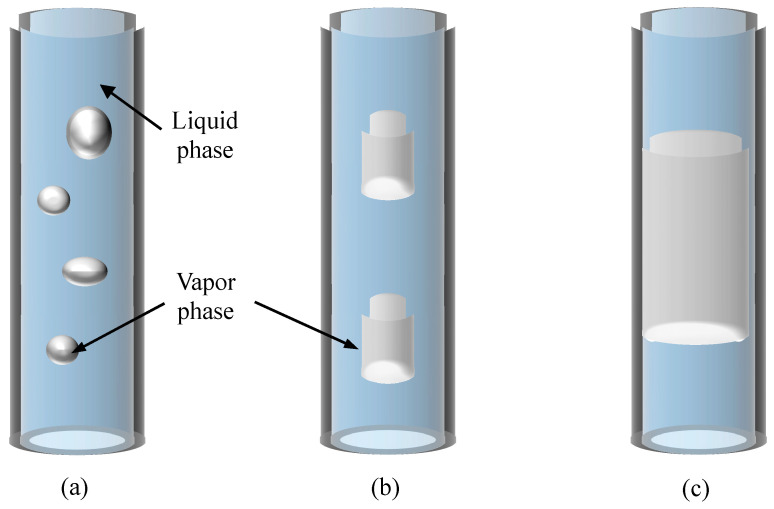
Schematic diagram of the main flow patterns of MPHPs: (**a**) bubble flow, (**b**) plug flow, and (**c**) annular flow.

According to current research [39], bubble flow, plug flow, and annular flow are the main flow patterns seen in MPHPs, as illustrated in Figure 9. The working fluid undergoes a phase transition during the evolution process as a result of heating in the evaporation portion, creating bubbles. Nucleate boiling takes over as the heat load steadily rises, creating a large number of bubbles in the evaporation section that quickly combine to generate plug flow. As the heat load grows further, the phase transition in the evaporation section becomes exceedingly intense, evaporating more working fluid. This eventually turns into annular flow when the fill ratio is low.

Qu et al. [40] experimented with PHP with different inner diameters using high-speed camera visualization technologies. The experiments showed that the evolution of flow patterns within the PHP underwent significant changes as the inner diameter of the pipe gradually decreased, in addition to observing the same primary flow patterns—bubble flow, plug flow, and annular flow—as those observed in SSPHPs. Interestingly, the plug flow in the SSPHP evaporation section (*D*_i_ = 2.5 mm, *D*_i_ = 2 mm) is created by the accumulation of many tiny bubbles. On the other hand, just one bubble’s growth can produce the gas plugs in the MPHP evaporation section (*D*_i_ = 1 mm, *D*_i_ = 0.5 mm). Additionally, it has been found that capillary wave annular flow forms more easily in micro-scale tubes than in small-scale tubes.

**Figure 10:**
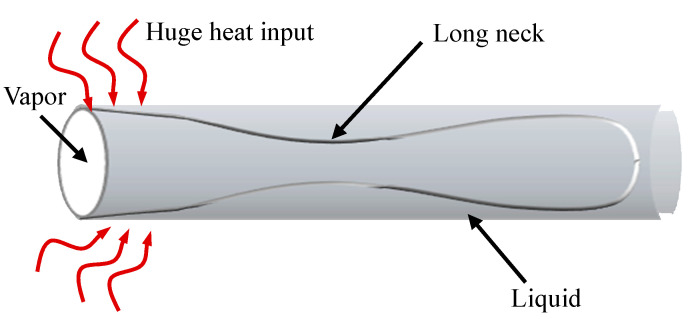
Schematic diagram of the injection flow.

In addition to the primary flow patterns described above, previously unreported flow patterns were observed within the MPHP. Using high-speed camera technology, Qu et al. [33] carried out visualization studies on a silicon-based MPHP with a hydraulic diameter of 394 μm and a trapezoidal cross-section. They found that an injection flow pattern similar to that previously seen in silicon micro-coolers appeared during the process of gas plug rupture at the tail end of the annular flow within the condensation portion of the MPHP channel and its subsequent development into plug flow. Sun et al. [32] fabricated a silicon-based MPHP with a trapezoidal cross-section and a hydraulic diameter of 357 μm. Using the dielectric fluid HFE-7100 as the working fluid, they conducted a visualization study employing high-speed camera technology. The results revealed transient C and bubble nucleation phenomena within the pipe. Additionally, they observed the annular flow in the evaporation section gradually forming a thinner gas plug ribbon as it flowed toward the condensation section. This ribbon eventually ruptured, creating an injection flow that progressively evolved into a bubble flow. More significantly, MPHPs with different hydraulic diameters was the subject of visualization investigations by Qu et al. [11,30]. Within a trapezoidal-section MPHP with hydraulic diameters greater than 352 μm, they saw nucleate boiling and injection flow. Nevertheless, there was no injection flow in the condensation portion when the hydraulic diameter was less than 352 μm. This happens as a result of the hydraulic diameter being too small to produce directed flow inside the tube. In the meantime, Liu et al. [27] observed injection flows in the adiabatic sections of silicon-based MPHP with rectangular cross-sections using high-speed camera technology. A schematic diagram of the injection flow is shown in Figure 10. Relevant studies have investigated the causes of injection flow. A visualization investigation on a MPHP with a hydraulic diameter of 411 μm was carried out by Wang et al. [25]. They found that the MPHP channel’s scale effects successfully stabilize annular flow, causing injection flow phenomena.

According to research by Wu et al. [41], surface tension creates a lateral pressure gradient at the gas–liquid interface when the curvature of the interface is non-uniform within microchannels (non-circular cross-section). Liquid flow is driven into particular areas (like bends) by this pressure differential, which results in localized thinning and rupture of the liquid film and, eventually, injection flow.

The link between microchannel diameter and injection flow frequency under various steam mass flow circumstances was examined by Quan et al. [42]. The findings show that the instability of the condensate layer rises with increasing steam mass flow or decreasing microchannel diameter, resulting in a greater injection flow frequency. Additionally, the injection flow occurs closer to the channel outlet.

**Figure 11:**
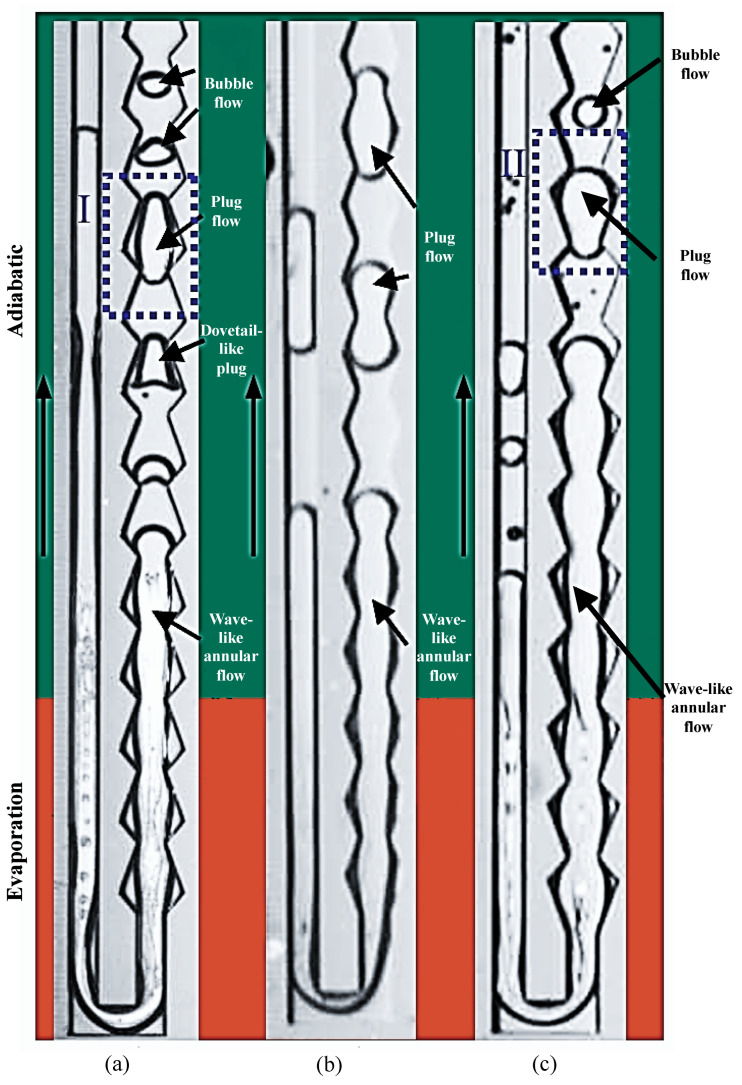
CDC-FPMPHP structures: (**a**) type A MPHP; (**b**) type B MPHP; and (**c**) type C MPHP. The image is sourced from the PhD dissertation of the corresponding author, Chao Wang [28]. Noted, the blue dotted boxes indicates different forms of plug flow, the vertical black long arrow indicates the flow direction.

Notably, Chen et al. [43] visualized the flat-plate micro pulsating heat pipe with asymmetric converging-diverging channels (CDC-FPMPHP for short) and the flat-plate micro pulsating heat pipe with uniform rectangular channels (UC-FPMPHP for short) using high-speed imaging techniques. Their results showed that the tube’s unique structure caused fluid disruptions and wall effects that produced characteristic “dovetail-like” plug flows and “wave-like” annular flows. Wang et al. [28] performed the same work and obtained results consistent with those described above (see Figure 11).

## 4. Experimental Study on Heat Transfer Performance of the MPHP

Heat transfer in the SSPHP principally relies on the pulsing motion of gas/liquid plugs. Numerous additional physical phenomena are unavoidably incorporated into this intrinsically complicated system as these heat pipes continue to shrink. For instance, because of the incredibly narrow channel diameters caused by miniaturization, surface tension has a much greater impact than other forces like gravity. This makes it challenging to directly use conventional two-phase flow theories and models because the flow and distribution patterns of gas–liquid phases differ significantly from those in the SSPHP. Second, the MPHP shows improved scale effects, wall effects, and interfacial effects in comparison to the SSPHP. These elements result in various flow characteristics, ultimately giving rise to distinctive heat transfer features. The MPHP’s heat transmission mechanism was investigated experimentally by Jo et al. [44]. According to the study, the main heat transfer mechanism in the MPHP is phase change heat transfer, also known as latent heat. Phase change heat transfer is not only the primary force behind the working fluid’s pulsation inside the pipe, but it also plays a major role in the MPHP’s total heat transfer performance. To assess the MPHP’s thermal performance, define the total thermal resistance $R=(T_{e}-T_{c})/Q$ as a thermal performance parameter. In this case, *T*_e_ stands for the average wall temperature of the evaporation section, *T*_c_ for the average wall temperature of the condensation section, and *Q* for the evaporator’s input power (the MPHP’s heat load).

### 4.1. Influence of Channel Structure Parameters

The main elements that must be decided upon during the design and production of the MPHP are the channel construction parameters, which serve as the basis for their heat transfer efficiency. Studies on this subject have been carried out by researchers both locally and abroad. The heat transmission efficiency of the MPHP with square and circular channels was investigated experimentally by Lee et al. [45]. According to the experimental results, the MPHP with a square channel shows less heat resistance than those with a circular channel under the same hydraulic diameter conditions. Their maximum permitted heat flux capacity increased by around 70%, demonstrating their enhanced thermal management capabilities. Additionally, the heat transmission limit rises as the hydraulic diameter grows for both circular and square channel MPHPs.

Kim et al. [46] performed heat transfer performance tests after etching reentrant cavities into the walls of an MPHP. MPHPs with reentrant cavities operate within the effective cavity region at moderate input powers, exhibiting lower thermal resistance than those with plain walls, according to an analysis of the thermal resistance of MPHPs with plain walls and reentrant cavities under various input powers. The voids also encourage early initiation and bubble nucleation within the MPHP.

Furthermore, Lee et al. [47] performed heat transfer performance tests and integrated a micro-post array (MPA) into an MPHP. They discovered that adding the MPA structure to the MPHP’s alternate channels raised the maximum permitted input power by 44% when compared to the MPHP without MPA.

In order to examine the heat transfer performance of surface features (rectangular pillar protrusions and hemispherical cavities) in the evaporation section of an MPHP under various operating conditions (working fluid, fill ratio, and input power), Kumar et al. [48]. The study found that the hemispherical cavity structures etched within the heat pipe actually encourage bubble nucleation and growth, in contrast to rectangular pillar protrusions that impede bubbles. As a result, the MPHP performs better in terms of heat transfer.

In recent years, the asymmetric channel design of the MPHP has gradually gained attention, with scholars both domestically and internationally conducting diverse research. According to research by Kwon et al. [49], the dual-diameter channel MPHP can provide a capillary pressure that is greater than the viscous pressure drop under some circumstances (where channel widths vary greatly). Because of this, the dual-diameter channel MPHP may demonstrate consistent thermal performance regardless of the inclination angle. The study simultaneously provided a figure of merit, which is defined as the ratio of capillary pressure difference to viscous pressure drop, based on standard heat pipe evaluation standards. For the alternating diameter MPHP to demonstrate angle-independent thermal performance, the figure of merit needs to be more than 2 × 10^5^.

The heat transmission capabilities of symmetric and asymmetric (dual-diameter channel) MPHPs under the same operating conditions were compared by Yang et al. [50] and Kim et al. [22]. Experiments have shown that MPHPs with asymmetric channels performs better at heat transmission than those with symmetric channels.

**Figure 12:**
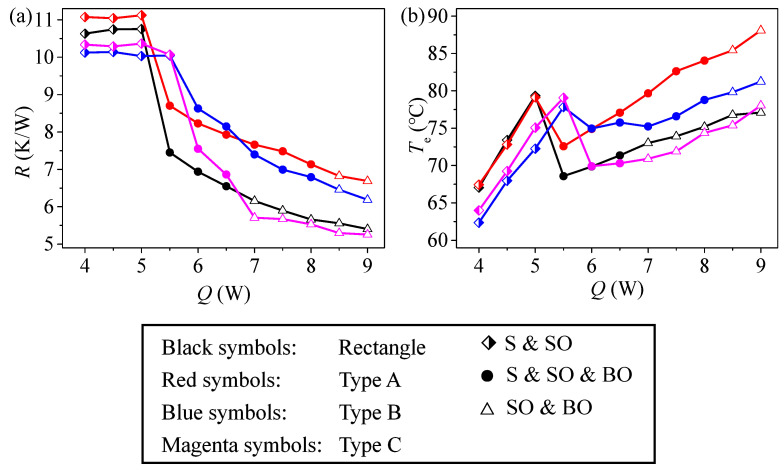
Thermodynamic performance comparison of various heterogeneous MPHP: (**a**) the thermal resistance, (**b**) the evaporation tem-perature [28].

As seen in Figure 12, Wang et al. [28] studied three non-uniform structures of variable cross-section channels with expansion and contraction (A: narrow at the top and wide at the bottom, B: uniform, C: narrow at the top and wide at the bottom). The results show that only the additional capillary driving pressure difference generated by type C can promote the movement of gas-liquid phase working medium to the condensation section while hindering the movement of working medium to the evaporation section, effectively strengthen forced convection evaporation/condensation and sensible heat transfer, reduce the temperature of the evaporation section, thereby reducing the thermal resistance and improving the heat transfer performance. Lim et al. [51] constructed a MPHP with several asymmetric channel layouts and examined how these layouts affected thermal performance under the same operating conditions. According to the study, a MPHP with asymmetric channel layouts (A2–6) outperformed those with symmetric channel layouts (S1) in terms of thermal performance and heat transmission limits. To improve thermal performance and operational range, the MPHP’s channels also need to show a high level of effective variety.

Huang et al. [52] created ratchet structures with different dimensional parameters (consistent with Figure 11) and compared them with symmetric channel MPHPs. The heat resistance of MPHPs with ratchet structures was consistently lower than that of symmetric MPHPs, according to an analysis of the various structures. This is explained by the ratchet structure, which improves heat transfer performance by increasing boiling and fluid turbulence inside the tube.

According to the aforementioned studies, a asymmetric MPHP often performs better in heat transfer than a symmetric MPHP. However, the aspect ratio between the channel cross-sectional width and depth, and the asymmetric ratios inside alternating channels, play a major role in how effective asymmetric structures are. The heat transfer properties of the MPHP with varied aspect ratios and asymmetry ratios under various input powers were examined by Jang et al. [26]. They discovered that heat resistance steadily dropped as the asymmetrical ratios grew, while the input power was at low to medium levels, and the aspect ratio stayed constant. Thermal resistance, however, increased as the asymmetrical ratios grew when the aspect ratio was high, and the input heat was relatively large. Furthermore, when input power grows from low to high, the ideal aspect ratio for obtaining oscillation, low flow resistance, and circulation changes from 3.3 to 2.5 for low asymmetry ratios. On the other hand, because there is less flow resistance at high asymmetry ratios, the ideal aspect ratio is always 2.5 regardless of the input power level.

Furthermore, it is impossible to ignore how other structural factors affect heat pipes’ thermal performance. The impact of condenser length on MPHP heat transfer performance at various condenser temperatures was investigated by Kim et al. [53]. The findings show that reducing the condenser length successfully lowers the evaporator temperature when the condenser temperature is in the lower range. On the other hand, expanding the condenser length correctly can actually lower the evaporator temperature when the condenser temperature climbs to the upper range. The MPHP was also found to have an ideal condenser section length.

The impact of the number of coils on the MPHP’s heat transmission limit was examined experimentally by Lee et al. [54]. They discovered that when the number of coils grows, the inclination angle’s impact on the maximum permitted heat flux density decreases. Regardless of the inclination degree, the maximum permitted heat flux density stays constant when the number of coils exceeds 20. According to research by Jun et al. [55], the closed-end MPHP only needs 10 coils to achieve thermal performance irrespective of tilt angle, in contrast to the closed-loop MPHP.

### 4.2. Effect of Inclination Angle (Gravity)

**Figure 13:**
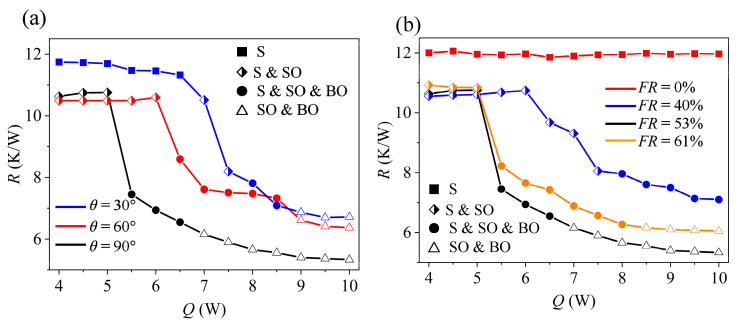
Thermal resistance under different operating conditions: (**a**) inclination angle; (**b**) fill ratio [28].

Researchers from all over the world have concentrated on examining the MPHP’s heat transfer performance under various inclination angles or gravitational fields since they frequently function at varied angles and spatial orientations in real-world applications. Qu et al.’s research [30,34] showed that even with the MPHP’s tiny tube segments, gravity still has a big impact on how they operate, thermal resistance progressively drops when the MPHP moves from an inclined to a vertical configuration. This law is also reflected in Wang’s researches [28], as seen in Figure 13a. Interestingly, as inclination angles grow, so does the MPHP’s heating power input, postponing the start of thermal transfer restrictions. This conclusion is also supported by the research conducted by Liu et al. [27].

Furthermore, two MPHPs were constructed by Gu et al. [13] to examine how gravity affected their heat transfer properties in microgravity. According to experimental findings, the MPHP performed better in heat transmission under reduced gravity than the same PHP under normal and hypergravity conditions. Additionally, this work offered the first proof that MPHPs may operate well in microgravity settings.

### 4.3. Effect of Fill Ratio

One of the main factors affecting the MPHP’s heat transfer performance is the fill ratio, which is the ratio of working fluid volume to the MPHP’s overall volume. According to current research, an extremely low fill ratio (FR < 20%) causes the evaporation section to have insufficient working fluid, which results in dry-out. On the other hand, a too high fill ratio (FR > 80%) reduces the gas–liquid phases’ oscillatory motion, which impairs heat transfer efficiency [56,57,58]. The experimental researches performed by Qu et al. [30] on the MPHP’s heat transmission performance under various fill ratios found that MPHP can only accomplish efficient pulsing heat transmission in the fill ratio region of 30% to 65%, where they also demonstrate superior heat transfer performance, according to the research. Additionally, it was discovered that PHP has an ideal fill ratio that corresponds to the lowest thermal resistance for every working situation.

Using acetone as the working fluid, Liu et al. [27] created a rectangular MPHP with a hydraulic diameter of 550 μm. They examined how well the MPHP transferred heat at various fill ratios. According to the study, the PHP failed when the fill ratio exceeded 74%. Wang [28] also conducted the experiment with the same system, and found that the lowest thermal resistance was attained at a fill ratio of 53%, as shown in Figure 13b. Using effective thermal conductivity as an assessment parameter, Kim et al. [37] examined the heat transfer performance of an asymmetric MPHP at various liquid fill ratios. They discovered that the effective thermal conductivity peaked at liquid fill ratios of 40–60% by examining the effective thermal conductivity of several working fluids.

As of right now, researchers both domestically and abroad largely concur that the ideal liquid fill ratio for the MPHP usually falls between 40 and 70%. The working fluid’s characteristics (such as surface tension and viscosity), the geometric structure (channel diameter and bend configuration), and the operating conditions (inclination angle and input power) all have an impact on the precise value.

### 4.4. The Influence of the Working Fluid

The working fluid’s physical characteristics, including surface tension, wettability, latent heat, specific heat capacity, and viscosity, all have a big impact on how well the MPHP transfers heat. Furthermore, scale effects become noticeable because of the MPHP’s small diameter, making the working fluid’s physical characteristics very important. In addition to impairing the MPHP’s heat transfer efficiency, using inappropriate operating fluids (ethanol and water) may even stop the PHP from starting up [30,34]. Working fluids should be chosen based on attributes like high (d*P*/d*T*)_sat_ values, low viscosity, low latent heat, and low surface tension, according to research by Khandekar et al. [59] and Qu et al. [30]. Hu et al. [60] used deionized water and self-rewetting fluid (SRWF) as working fluids to compare the MPHP’s heat transfer capabilities. They discovered that SRWF has the special ability to increase surface tension with temperature, in contrast to deionized water. This feature lowers heat resistance by increasing the working fluid’s C efficiency.

An MPHP with a 50% liquid fill ratio and a hydraulic diameter of 667 μm was manufactured by Kim et al. [31]. They looked into how well the MPHP transferred heat using various working fluids. The results of the trials showed that ethanol, FC-72, HFE-7000, R-245fa, and R-134a were the working fluids with the highest to lowest thermal resistance.

Nanofluids are a new working fluid for PHP with special benefits that can improve PHP’s heat transfer efficiency. They are now a major field of study for academics both domestically and abroad. An MPHP with a hydraulic diameter of 508 μm was created by Jahani et al. [61]. Water and nanofluids (ferrofluid and silver nanofluid) were used as working fluids in experiments and comparisons. According to the experimental investigation, MPHPs that use nanofluids as working fluids perform better at heat transmission and have lower thermal resistance than those that use water as the working fluid.

### 4.5. Other Influencing Factors

Apart from the aforementioned key impacting elements, it is important to consider a number of secondary aspects. For example, steam distribution [39], surface treatment [62], and the frequency and amplitude of fluid plug pulsations inside the tube [63,64,65] can all successfully increase the MPHP’s heat transfer capacity.

## 5. Conclusions

One of the most important technologies for effective heat dissipation in electrical devices is the MPHP. The scale effects, interfacial effects, and wall effects inside the microchannels of the MPHP become more noticeable compared to the conventional SSPHP. More unique patterns and phenomena are seen in the internal pulsing flow of a gas–liquid two-phase fluid, resulting in more diverse heat transfer properties. With an emphasis on both flow properties and heat transfer performance, this paper offers a thorough analysis of experimental research on MPHPs. The following are the particular conclusions:

(1) High-speed camera technology is the most widely used visualization technique, but it requires either a metal-based/silicon-based plate-type MPHP or a specifically manufactured tubular MPHP made of transparent glass material.
(2) The flow pattern inside the tube changes as the input power rises, starting as a bubble flow and progressing to a plug flow and annular flow. Additionally, the motion pattern of the working fluid inside the tube progressively changes from SO to LO to C.
(3) The C may stop under the impact of variables, including filling ratio, working fluid parameters, and hydraulic diameter, resulting in sporadic S occurrences inside the pipe. The motion mode influences the flow pattern inside the pipe, and injection flow is visible in areas like the condensation zone.
(4) Channel structure, inclination angle, working fluid physical characteristics, and fill ratio are some of the variables that affect the MPHP’s heat transfer performance. In particular, the MPHP with asymmetric channel configurations (such as alternating diameters) shows better heat transfer performance and less thermal resistance than symmetric channel structures. High (d*P*/d*T*)_sat_ values, low viscosity, low latent heat, and low surface tension working fluids can efficiently promote C inside the tubes, improving the MPHP’s heat transfer efficiency. Furthermore, when placed vertically and running at a moderate fill ratio (40–70%), the MPHP demonstrates greater heat transmission characteristics.

## Data Availability

No new data were created or analyzed in this study. Data sharing is not applicable to this article.

## Abbreviations

The following abbreviations are used in this manuscript:

MPHP	Micro pulsating heat pipe
SSPHP	Small-scale pulsating heat pipe
PHP	Pulsating heat pipe
FPPHP	Flat plate pulsating heat pipe
SO	Small oscillation
LO	Large oscillation
C	Circulation
S-O	Stable oscillation
BO	Bulk oscillation
S	Stop-over
P	Pulsing flow
UO	Unstable oscillating flow
LAOP	Large-amplitude oscillation phase
SAOP	Small-amplitude oscillation phase
CDC-FPMPHP	Flat-plate micro pulsating heat pipe with asymmetric converging-diverging channels
UC-FPMPHP	Flat-plate micro pulsating heat pipe with uniform rectangular channels
MPA	Micro-post array
FR	Fill ratio
